# Clinicopathological, immunohistochemical, molecular-genetic and risk profiles of gastrointestinal stromal tumors in a cohort of Sudanese patients

**DOI:** 10.4314/ahs.v23i1.47

**Published:** 2023-03

**Authors:** Nazik Elmalaika Husain, Ihsan Mohamed Osman, Ahmed Khalid, Ali Abdel Satir, Robert Stoehr, Abbas Agaimy

**Affiliations:** 1 Department of Pathology, Omdurman Islamic University, Sudan, nazikhusain@gmail.com,; 2 Department of Pathology, Faculty of Medicine, Alzaiem Alazhari University, Khartoum North, Sudan; 3 Dept. of Oncology, Khartoum Oncology Hospital, Sudan; 4 Histo Center, Khartoum, Sudan; 5 Institute of Pathology, University of Erlangen, Germany

**Keywords:** Gastrointestinal stromal tumour, GIST, risk stratification, malignant behaviour, risk assessment, tumour rupture, metastasis, Imatinib, genotyping, KIT, DOG1

## Abstract

**Background:**

Determining the risk of malignant behaviour and mutational status of gastrointestinal stromal tumours (GISTs) guide the management decision and allow optimal individualized patient treatment.

**Objectives:**

To determine clinicopathological, immunohistochemical (IHC), risk and KIT mutational findings of GISTs in Sudanese patients.

**Methods:**

Histological slides were reviewed, IHC for DOG-1 and CD117 performed and hotspot KIT mutations examined. The risk group was assigned using combined risk criteria.

**Results:**

21 of the 36 patients (58.3%) were males (mean age, 54.83 ±12.57; range, 26-71). Abdominal pain and mass were the most frequent symptoms. Mean tumor size (±SD) was 11.6(±5.82) cm. Either CD117, DOG1 or both were positive in all cases. Using risk criteria, 33.3% (n=12) were clinically malignant at presentation, 13.9% (n=5) high risk, 16.7% (n=6) intermediate, 27.8% (n=10) low risk and 2.8% (n=1) very low risk. Sixteen of 23 (70%) tested cases had KIT (14 exon 11 and two exon 9) mutations. Six tumors were wild type. Exon 11 deletions (p.I563-L576 del and p.V559-N566delinsD) significantly correlate with disease recurrence (p-value: 0.028).

**Conclusions:**

Sudanese patients with GIST tend to present late. Nearly half of them correspond to the malignant/high-risk category. The frequency of KIT mutations (79.31%) is in line with the literature.

## Introduction

Although rare, gastrointestinal stromal tumours (GISTs) are the most common mesenchymal neoplasms of the digestive tract, with an estimated frequency of 20%-30% of all soft tissue sarcomas and an overall annual incidence of 10-20 per million^1^,^2^.

Identification of GIST became more critical after the availability of effective selective (imatinib mesylate/Glivec) or multikinase (sunitinib) inhibitors for the treatment of unresectable and metastatic tumours^3^ as well as a standard adjuvant treatment for patients with high risk for relapse after total (R0) resection. Thus, determining the risk of malignant behaviour or recurrence guides the management decision.

Different risk stratification systems have been suggested for GISTs. The first risk classification scheme in the imatinib era was proposed by an expert consensus workshop held at the National Institute of Health (NIH) in Bethesda in 2001 (Fletcher et al.) and was based solely on tumour size and mitotic count^4^. Although this system became popular among oncologists and pathologists, it has some limitations^5^, as it tends to over-classify larger mitotically inactive gastric GISTs and under-classify some duodenal or rectal tumours because it does not include the anatomic site and presence of tumour rupture^5^.

A few years later, and based on the Armed Forces Institute of Pathology (AFIP) database, Miettinen et al. added the anatomic site (gastric vs non-gastric) as an additional risk criterion^6^. They defined eight risk subgroups (1 to 6b) and gave the risk of malignancy in percentage based on long-term follow-up studies. This enabled reliable decisions regarding adjuvant therapy^5^. The ESMO and NCCN guidelines recommended use of these “AFIP” criteria. In addition, the AFIP system recognized small mitotically inactive tumours as benign (nor risk)^6^.

A modification of the NIH system with the inclusion of the anatomic site and tumour rupture (spontaneous or at surgery) was proposed by Joensuu^7^. Additional criteria proposed by several groups includes non-radical resection^6^ and a “clinically malignant (metastatic or widely invasive) group”^8^. An integrated approach combining all these criteria seems to be more promising in identifying those who need tyrosine kinase inhibitor (TKI) therapy^9^. Overall, 16 categorical and seven continuous risk stratification systems have been published (nomograms, heat/contour maps, and mathematical models are examples of the “continuous variables”-based systems)^2^.

GISTs are thought to originate from or differentiate similar to the gut pacemaker cells, the interstitial cells of Cajal (ICC)^10^. Identification of recurrent gain-of-function mutations in the KIT proto-oncogene^11^ and later of PDGFRA as a driver of GIST (overall in 80-85% of cases) led to the rationally-based use of Imatinib. Imatinib is a TKI with activity against KIT, PDGFRA and bcr/abl^12^.

Around 75% of KIT mutations in GISTs affect exon 11 (the intracellular juxta membrane domain of the receptor) and result in spontaneous (ligand-independent) receptor activation 1. Tumours with exon11 mutations respond to the standard imatinib dose (400 mg/day). On the contrary, those with exon 9 mutations (extracellular domain) need a double dose. Mutational analysis is a valuable adjunct for definitive diagnosis and better treatment choices for these patients and those with inconclusive diagnoses^13^.

In Sudan, GISTs account for 0.49%, 0.57%, and 0.74% of the new cancer cases registered during three sequential years in the Khartoum Oncology Hospital (KOH) database. However, published population-based studies are lacking, only insufficient institution-based studies are available, and no data on molecular analyses have been published to date. For example, in a single histopathology laboratory, there were only 5 GIST cases among 1958 malignancies from different body organs from 2000 to 2004^14^.

This study aimed to describe the clinical, histopathological and immunohistochemical features of GISTs in a cohort of Sudanese patients attending KOH. It also stratified the cases for the risk of progression, assessed the quality of histopathological GIST reporting during the study period and tested a sub-cohort for KIT mutations. Up to writing this manuscript, no data is available on the mutation status among Sudanese patients with GIST.

## Methods

### General characteristics

The study patients were recruited from the Glivec International Patient Assistance Program (GIPAP) Clinic in Khartoum Oncology Hospital (KOH; previously named “Radiation & Isotope Centre /RICK”), Khartoum, Sudan. KOH is the main oncology centre in Sudan receiving cancer cases from different regions of the country. It is the country's only oncological (non-surgical) GIST treatment centre.

The study included Sudanese patients with a histological diagnosis of GIST for two consecutive years (24 months) in KOH and agreed to be enrolled. Patients without accessible tissue blocks, with revised diagnosis after histological reassessment, or who have no sufficient tissue for further assessment were excluded.

Data regarding demographic characteristics, presenting symptoms and duration of symptoms were collected using a predesigned tested questionnaire and direct interview of patients after signing a written informed consent. The clinicopathological parameters, including tumour size, site, and presence of metastasis at diagnosis, were collected from the patient's medical records. Pathological features, including mitotic count and histologic type, were determined from Haematoxylin and Eosin (H&E) stained slides.

Mitotic figures were calculated in consecutive fields in the most mitotically active area of the tumour, considering a total area of 5mm^2^ according to the current ESMO guidelines. Also, counting was extended to conventional 50 HPFs for comparison purposes.

### Immunohistochemistry

Immunohistochemistry (IHC) was done for DOG1 (monoclonal DOG-1 antibody, 1:200, Novocastra, Newcastle, UK), CD117 (polyclonal antibody, 1:200, DAKO, Hamburg Germany) and PDGFRA (monoclonal anti-PDGFRA antibody (#3164, dilution: 1:100; Cell Signalling Technology, USA). For PDGFRA, only para nuclear dot-like/ Golgi staining (with or without membranous staining) was considered specific 15. IHC was performed on 3-µm sections using a fully automated system (“Benchmark XT System”, Ventana Medical Systems Inc.)

### Molecular analysis

Based on morphology and tumour site, mutation testing was performed either for KIT exon 11 (if spindled gastric), KIT exon 9+11 (intestinal) or PDGFRA (epithelioid gastric). A total of thirty cases were tested using this approach (29 were tested for KIT exon 11 and/or nine and one for PDGFRA exon 18) as described previously^9^. First, genomic DNA was extracted from serial sections (5µm) of FFPE tissue blocks. Then, after deparaffinization, areas of interest were macro-dissected manually. According to the manufacturer's instructions, DNA was prepared using the Maxwell® 16 MDx Research Instrument (Promega, Mannheim, Germany).

DNA extraction, Polymerase chain reaction (PCR) and sequencing of KIT and PDGFRA were carried out using the methods and primers previously described by Daniels et al. 16.

Data were processed using Statistical Packages for Social Sciences, version 23.0. Descriptive statistics, a student t-test, Pearson Chi-square and Fisher's Exact tests were used where appropriate. A P-value of <0.05 was considered statistically significant.

Succinate Dehydrogenase B (SDHB) status was determined using a polyclonal anti-SDHB antibody (dilution, 1:200, Sigma-Aldrich). A complete lack of granular cytoplasmic staining in the neoplastic cells was considered deficient compared to granular solid routine cytoplasmic staining (proficient). Strong expression in the endothelial cells and other normal stromal cells in the background d was required for assessable staining.

## Results

### General demographic and clinical features

One hundred and fifteen patients were seen at KOH during the study period. Two were excluded for being non-Sudanese, and one died before the initiation of the study. Of the remaining 112 Sudanese patients, 88 were interviewed and signed a written informed consent (78.57% response rate). The Formalin-Fixed Paraffin-Embedded (FFPE) tissue blocks were collected from different histopathology laboratories (n=17) inside and outside Khartoum. Fifty-three cases had available blocks and were revised by an expert pathologist (A.A.). Of these, 13 were excluded as non-GIST (24.53%) (Table 1), and four had no sufficient tumour tissue for further analyses. Thus, a total of 36 patients represents the base of the current study.

**Table 1:** Revised non-GIST cases in the period Jan 2014-Dec 2015

Serial No.	Age	Gender	Site	Diagnosis after revision
1	23	F	Ovary	Most likely fibrosarcoma
2	67	M	Abd mass?	De-differentiated Liposarcoma
3	60	M	Spinal cord L3,4	Non-GIST, unclassified
4	48	M	Rectum	Neuroendocrine tumour
5	58	M	Mesentery	Leiomyosarcoma
6	60	M	Rectum	Melanoma
7	60	F	Sigmoid colon	Melanoma
8	70	F	Gastric body + oesophagus	Lymphoma
9	62	M	Small intestine	Leiomyosarcoma
10	60	F	Small bowel mesentery and pelvic cavity	Melanoma
11	45	F	Liver metastasis with unknown origin	Metastatic thyroid carcinoma
12	23	M	Rectum	Adenocarcinoma
13	37	F	Small intestine	B-cell NHL

Table 2 shows the demographic distribution and clinicopathologic features of patients. Any interviewed patients did not report a first or second-degree family history of GIST.

**Table 2:** Demographic and clinicopathological characteristics of the study cohort (n=36)

Characteristic	Category	Per cent
Age	Range: 26–71 Mean (±SD): 54.83(±12.57) M: F ratio=1.4:1	-
Gender	Males:21 Females: 15	58.3 41.7
Geographic distribution	North	22
	East	14
	West	42
	Gezira	19
	Blue Nile	3
	Total	100
Primary tumour site	Gastric	63.9
	Small intestine	27.8
	Colon	2.8
	Oesophagus	2.8
	Not specified	2.8
	Total	100.0
Number of mitoses /50HPF	<5	77.8
	6–10	5.6
	>10	2.8
	Not enough to count 50 HPF	13.9
	Total	100
Diagnostic procedures used	Upper or lower GI endoscopy	36
	U/S	22
	CT scan	36
	MRI	3
	Others	3
	Total	100
Specimen types	Endoscopic biopsy	11
	Image-guided needle biopsy	20
	Surgical excision	69
Histologic types	Spindle	69
	Epithelioid	28
	Mixed	3
	Total	100
The outcome	Alive	69.4
	Died	25
	Unknown	5.6
	Total	100

Abdominal pain and swelling were the most frequent symptoms. Symptom duration ranged from 1 to 120 months (mean ±SD of 26.93±32.46 months).

26 of the 36 patients (72.2%) underwent surgical intervention that varies depending on the tumour's location and extent (wedge resection, partial or total gastrectomy, segmental resections or multivisceral resection).

22.2% (n=8) received neoadjuvant Imatinib therapy. The remaining 77.8% (n=28) received adjuvant Imatinib therapy for 1 to 48 months. Applied dose was 400 mg/day (dose was escalated to 600 mg/day in three patients).

Most tumours were gastric (63.9%, n=23), and 27.8% (n=10) originated in the small intestine.

The 2-sided Pearson Chi-square test between the number of mitoses in 5mm2 vs. 50HPFs shows no superiority for either screened area.

Unspecified (whether spontaneous or surgical) tumour rupture was described in 19.4% (n=7) and was not mentioned 52.8% (n=19) of the cases. Invasion of the surrounding structures was present in 30.6 % (n=11), absent in 30.6% (n=11) and not reported in 16.7% (n=6) of resected tumours.

Metastases were present at presentation in 27.8% (n=10) of cases: hepatic in 19.4% (n=7) and peritoneal in 8.3% (n=3) of these cases. Metastasis status was unknown in 16.7% (n=6) of the cases. Three of the seven cases with tumour rupture also had liver metastasis, but none presented with peritoneal deposits.

Four and two out of the seven cases with tumour rupture have tumour size >10 and 5-10 cm, respectively. However, this association is not statistically significant (p-value 0.50).

### Pathological findings

Tumour size was reported in 33 cases (4 to 30 cm with a mean (±SD) of 11.6(±5.82) cm)).

**Figure 1:**
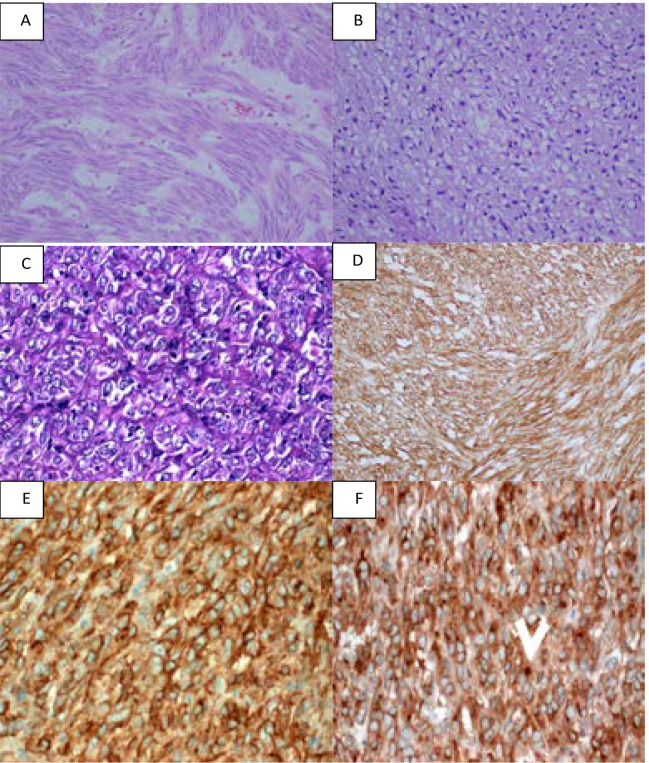
A: Intestinal GIST with spindle cell histologic pattern (H&E, X400). B: Gastric epithelioid GIST exhibiting signet ring-like vacuolated cytoplasm (H&E, X400). C: Epithelioid GIST showing monotonous epithelioid morphology with fine reticular fibrosis resulting in organoid nesting pattern (H&E, X400). D: CD117 Immunohistochemical (IHC) expression in spindle cell GIST (CD117 IHC, X400). E: Epithelioid GIST showing strong membranous positivity for DOG1 (DOG1 IHC, X400). F: Gastric epithelioid GIST showing cytoplasmic and characteristic strong Golgi-pattern (arrow) positivity for PDGFRA (PDGFRA IHC, X400).

Cystic change was reported in 27.8% (n=10), while 33.3% (n=12) of the histopathology reports didn't report the tumor consistency. The majority (69.4%, n=25) of tumours were of spindle cell histological type, but different patterns were seen (Figure 1A-C).

### Immunohistochemical findings

Of studied cases, CD117 was positive in all (Figure 1D), CD34 in 61.1% (n=22), and DOG1 in 78.79% (n=26) (Figure 1E). Other immunohistochemical markers were used to exclude other diagnoses in 30.6% (n=11) and included SMA, S100, Desmin, and others. PDGFRA was positive in one case and showed a Golgi pattern (Figure 1F).

### Risk stratification results

Risk stratification of the studied GIST cases was shown in Table 3 according to Fletcher et al. (so-called NIH criteria), Miettinen & Lasota (so-called AFIP criteria), and the integrated system. In addition, a comparison between the integrated risk group and AFIP and NIH groups is illustrated in Figure 2 (P. value 0.000 for each).

**Table 3:** Compared risk stratification of the studied GIST patients according to the NIH, AFIP and the integrated risk criteria

Risk group	NIH risk group		AFIP risk group		integrated risk group	
	Freq.	%	Freq.	%	Freq.	%
Very low risk	5	13.9	1	2.8	1	2.8
Low risk	0	0	12	33.3	10	27.8
Intermediate risk	13	36.1	7	19.4	6	16.7
High risk	15	41.1	13	36.1	5	13.9
High risk (clinically malignant)	NE	NE	NE	NE	12	33.3
Not classified	3	8.3	3	8.3	2	5.7
Total	36	100	36	100	36	100

NE: not existing.

NIH risk group -unlike other risk criteria- correlates significantly with the overall survival (p. value 0.016), but not with mutation type.

### Imatinib therapy, patients' outcome and prognostic parameters

Patients were followed for (mean ±SD) of 30.33 ±14.02 months clinically and by six-monthly radiologic studies. 72.2% (n=26) of the patients used Imatinib regularly. Out of the followed patients (n=34), 36.1% (n=13) showed evidence of progression and 16.7% (n=6) of recurrence during the follow-up period. By the end of the follow-up period (maximum 50 months), 25(69.4%) were alive, and 9 (25%) died of unspecified cause.

The patient's age was significantly related to the regularity of imatinib use, with older age groups (more than 46 years) tending to be more regular (P-value 0.026).

A significant correlation was noted between the patient's gender and tumour necrosis (P-value 0.010), tumour haemorrhage (Pearson Chi-Square: 0.008, Fisher's Exact Test (1-sided): 0.010), tumour recurrence (P-value 0.028), outcome ‘alive or not’ (P-value 0.030) for the benefit of females.

### Molecular genetics findings

23 of 29 cases were successfully assessed for mutations in KIT hotspots in exon 11 and 19/21 for mutations in exon 9.

14/19 cases (73.68%) were exon 11 mutation-positive, and five (26.31%) were wild-type (four cases were not sequenced). Table 4 shows KIT Exon 11 mutations detected in the successfully assessed GIST cases.

**Table 4:** KIT Exon 11 mutations detected in the successfully assessed GIST cases

Mutation	Number	Per cent
p. H580_K581_insPTQL	1	7.14
p. L576 del	1	7.14
p. V560 del	1	7.14
p. V560_L576 del	2	14.29
p. W557_K558 del	1	7.14
p.I563_L576 del	1	7.14
p.K550_K558 del-insL	1	7.14
p.K550_p. K558 del	2	14.29
p.V559_N566 del-insD	1	7.14
p. W557R	1	7.14
p.Y553_Q556 del	1	7.14
Not identified	1	7.14
Total	14	100

Three of the five tumours with exon 11 mutations were also tested for KIT exon 9 mutations; none had a mutation in line with the mutually exclusive nature of exon 11 and exon 9 KIT mutations in GIST. One case with wild-type exon 9 was not assessable for exon 11. Three tumours were not assessable for both exons.

Most exon 11 mutations clustered in the proximal region of the exon at codons 553-560. Only two tumours harboured a distally located mutation at L576del, and most (64.3%) were deletions. Nine of the 15 mutations (60%) were deletions, while 2/15 (13.33%) harboured deletions and insertions. Two (10.53%) exon 9 mutations have been detected in the sub cohort with successful molecular testing for exon 9 mutations. Both were from the small intestine and they represented the p.Y503_F504insAY duplication known to require higher Imatinib dose (800 mg/d). The overall KIT mutation is 79.31%.

Exon 11 deletion mutations (pI563-L576 del, p.V559-N566delinsD and PI563-L576 del) significantly correlates with recurrence (p. value: 0.028). The only case examined for exon 18 PDGFRA proved to be wild-type. Only one tumor was succinate Dehydrogenase B (SDHB)-deficient. It was a gastric GIST in a 36-year-old male.

## Discussion

This study on a cohort of 36 Sudanese patients diagnosed and treated for GIST showed similar age distribution (range: 26-71 years) as reported in other published studies 17, 18. However, the mean age (54.8) is slightly lower than the reported mean age in Lebanese patients (62.8 ± 12.8 years) 19 and also lower than the mean age in three European population-based studies (mean 66 to 69 years)^20^. 18.

In the current study, the drug (Imatinib) adherence was significantly related to the patient's age, with older ages tend to be more adherent to their drug (P-value 0.026). A significant correlation was noted between the patient's gender and tumour necrosis (P-value 0.010), tumour recurrence (P-value 0.028) and outcome (P-value 0.030) for the benefit of females. Some published studies reported younger age (<50 or 40 years) and female gender to be significantly linked with a more favourable prognosis in GIST 21, while others did not. Hatipoğlu et al. showed a significant correlation between survival and age but not gender 22. On the other hand, Molinas Mandel et al. did not consider age as a prognostic indicator 23. The good outcome of the female patients may be due to the increased commitment to therapy, or they may seek health care early. Nevertheless, hormonalactors cannot be excluded.

Most (72.2%) of the studied patients came from different Sudan states since Khartoum Oncology Hospital (KOH) is the only place to seek management of GIST in Sudan. In addition, tyrosine kinase inhibitors; Imatinib and sunitinib are made available free of charge to all patients with CD117-positive GIST through the GIPAP (Glivec International Patient Assistance Program). The GIPAP was established by Novartis Oncology in 2002, in partnership with MAX Foundation providing medicines by full donation to properly diagnosed patients in countries around the world without government or private reimbursement and who cannot pay for the medication introduced to Sudan in 2003). Out of the 53 patients referred to KOH within two years and reviewed in this study, 13 (24.53%) were excluded for being non-GIST, indicating over diagnosis of GIST. This point is particularly relevant, as these cases were diagnosed in the study period 2014-2015 and did not represent old diagnoses preceding the KIT era.

The studied patients belong to different Sudanese tribes. However, four tribes (Bedairia, Fur, Gaaleen and Nuba) show slightly higher frequencies (8.3% for each), but not to the level of statistical significance. Strikingly, a large group of patients (41.7%) are from the western states, an observation that necessitates further studies. In the present study, the first or second-degree family history of GIST was not reported by any of the interviewed patients. This is in keeping with the sporadic nature of GIST in adults.

The presenting symptoms of GISTs vary greatly. GISTs can be asymptomatic (discovered incidentally) 24 or present with an acute emergency like intestinal obstruction due to strangulated inguinal hernia 25. In addition, it can be associated simultaneously with another cancer 26, 27 or, rarely, other medical conditions such as renal transplant 28. In the current study, similar to an Indian study 41, abdominal pain and swelling were the most frequent presenting symptoms. However, some cases present with intestinal obstruction; one was discovered during the investigation for unrelated reasons.

The duration of symptoms has a wide range in this study (1 to 120 months with a mean (±SD) of 26.93±32.46 months). At the time, small incidentally detected tumours were becoming recognized increasingly, and a few developed into clinical GIST 29, 30. The mean tumour size of Sudanese patients is more than 10 cm. Sudanese patients seek medical advice late; this may be partially explained by the denial of the truth of having cancer, which represents a social stigma to some, or the shyness and increased pain tolerance of others. Poverty and lack of easily accessible medical services may contribute largely to this delay. Additionally, some of the patients do seek alternative medicine first.

Early diagnosis of GISTs is essential for better treatment outcomes and survival rates /span> 31. The recent advent of high-resolution radiological techniques, including CT scans, MRI and EUS, helped diagnose even asymptomatic small lesions 32. In the present study, CT scan and endo- or colonoscopy were the most commonly used diagnostic procedures (36.1%, n=13 each), an observation that is similar to other studies 31. Interestingly, it has been reported that certain CT features such as large tumour size, mixed growth pattern, enlarged vessels feeding or draining the mass and the solid enhancing component can significantly predict the malignant behaviour of the disease 33–35. However, risk stratification still represents the gold standard for prognostication in GIST.

The definitive management for localized primary GIST (>2cm) is complete surgical excision 36 specially in younger patients and it is associated with better overall survival (OS) and GIST specific survival (GSS) when compared with surgical management in older patients (OS: 91.1% vs 77.2%, P = .01; GSS: 91.8% vs 78.0%, P = .008) 37. Even in metastatic GISTs, surgery proved to be effective^38^. Nevertheless, Sato et al. found no significant difference in the 5-year overall survival rate between patients who underwent R0/R1 and R2 resection (71.4 vs 68.6 %) 39. In this study, 72.2% (n=26) underwent surgical intervention that varies greatly depending on the anatomical location of the tumour and its extent; therefore, the surgical excision specimens predominate. None of the surgical interventions in the current series was done through laparoscopy, which proved beneficial over open resection in terms of less blood loss and shorter hospital stay 40.

The definitive diagnosis of GISTs as recommended by the European Society for Medical Oncology (ESMO) and the National Comprehensive Cancer Network (NCCN) is based on tumour morphology and immunohistochemistry^41^–^43^. Therefore, surgical excision or a tumour biopsy is necessary for histopathologic examination since imaging modalities cannot diagnose GIST conclusively.

This survival rate (69.4%) is lower than that reported by other studies (93%) 46; late presentation with large tumour size (mean 11 cm) and irregular imatinib use (only 72.2% (n=26) of the patients used Imatinib regularly) may partially explain this unfortunate outcome. Patrikidou et al. reported a similar overall survival of 69.1% at five years on a median follow-up of 73 months.

In this study, the percentage of patients who were stratified as ‘high risk’ and/or clinically malignant (i.e., eligible for Imatinib treatment) was high using the integrated risk stratification systems (47.2%) compared to the NIH (41.7%) and the AFIP (36.1%) criteria, respectively. This is not in keeping with a comparable study by Schmieder et al. They assessed five well-known risk stratification systems (Fletcher, Miettinen, Huang, Joensuu, and TNM classification) on 558 patients with localized respectable GISTs. They found no significant difference between the five systems in their ability to stratify patients into high or low-risk groups or predict patient outcomes. They highlighted the need for more precise tumour and patient-related criteria for better stratification and identifying GIST patients who will benefit from adjuvant imatinib therapy^47^.

Moreover, the large tumour size at presentation (a mean (±SD) of 11.6(±5.82) cm) together with the presence of metastasis at the presentation in 27.8% of the studied group signifies a late presentation of Sudanese patients. Moreover, in this study, the integrated risk criteria showed a 14.7% and 17.6% greater tendency to enrol patients in Imatinib treatment than the NIH and AFIP, respectively. Therefore, the integrated risk stratification system may be more suitable for a low-income country, such as Sudan, where most people present late with distant metastasis.

On the other hand, the use of NIH, AFIP, and integrated risk stratification systems showed that 51.2%, 56.1% and 41.5% of the studied patients were using Imatinib without a justifiable indication. Therefore, it is worth mentioning that a more significant fraction of patients has been using Imatinib without a reasonable indication. Thus, awareness of the strict indications for Imatinib therapy and adherence to established guidelines is still to be significantly improved, as observed from the high frequency of Imatinib use irrespective of low-risk profiles of patients.

Despite the limited sample size, this study drew the attention of oncologists in charge of treating GISTs patients in Sudan to the importance of using risk stratification criteria in terms of considering adjuvant therapy, optimizing treatment (duration), and appropriate patient counselling with the additional benefits of preventing drug side effects and keeping the resources. The current study used three criteria, NIH, AFIP and the integrated risk stratification criteria, to determine the malignant potential of GISTs. Accordingly, patients were re-evaluated and treated as to the current standard of care. This was applied to the study group; all patients were referred to KOH. As a result, Imatinib was stopped for those in the low-risk group, those with high risk will use it for three years, and patients with a metastatic tumour will receive it indefinitely. It also highlights the importance of adherence of pathologists to the guidelines in diagnosing GIST and the usefulness of the multidisciplinary approach (clinical, operational and radiological findings) to improve the clinical practice and patient outcomes in developed countries 48.

The frequency of exon 11 mutations and kit mutation in this study is similar to other international studies 49, 50, 51. In the current study, most exon 11 mutations clustered in the proximal region of the exon at codons 553-560. Only two tumours harboured a distally located mutation at L576del, and most (64.3%) were deletions. Nine out of the 15 mutations (60%) were deletions, while 2/15 (13.33% harboured both deletions and insertions, and these exon 11 deletion mutations (pI563-L576 del, p.V559-N566 delinsD and PI563-L576 del) significantly correlate with recurrence (p-value: 0.028).

Furthermore, two (10.53%) exon nine mutations have been detected in the sub cohort 13 with successful molecular testing for exon nine mutations; both were from the small intestine. They are at p. Y503_F504insAY. This mutation type (duplication) is known to be less sensitive to Imatinib in the usual dose of 400 mg/d; instead, patients with this mutation require a double dse of Imatinib (800mg/d). Interestingly, Mutations in exon nine are significantly associated with the primary tumour site; they tend to be detected in intestinal GISTs (p. value 0.007) and associated with adverse outcomes (0.026).

Several factors affect the prognosis following imatinib treatment. Published literature reported primary and secondary imatinib resistance. Secondary KIT mutations are known to arise most commonly in exons 13 (the cytoplasmic ATP-binding domain, ABD) or exons 17 (the activation loop, AL). In contrast, primary KIT mutations predominantly affect the juxtamembrane domain encoded by exon 11. Secondary resistance or progression under correct treatment dosage can be caused by the occurrence of secondary mutations, mainly in exons 13, 17 of KIT or exons 14,18 of PDGFRA (including D842V mutation)^52^, ^53^. However, they are detectable in 50% of cases only. Other mechanisms include alternative molecular mechanisms to escape the drug, like receptor amplification, conformational changes, or epithelial-mesenchymal transition (EMT). CD34+KIT low stem cells for GIST, cancer cell quiescence, and altering the metabolic phenotype of GIST through reactive oxygen species (ROS0 and hypoxia-inducible factor-1α (HIF-1α) can play a role 54–56. In this study, we have no data on secondary mutations of the recurrent cases. There was no available tissue for testing; surgery of the recurrent tumour or re-biopsy was not done for the patients with recurrence.

Furthermore, mutation testing for secondary resistance or recurrence under treatment is not considered a clinical practice yet 53. Drug non-compliance (non-adherence), suboptimal dose, and inter-ethnic differences in imatinib pharmacokinetics and dosing should be sorted and verified first 57,58. Not to forget that other factors, including gastrointestinal bleeding, Ki67 index, prognostic nutritional index (PNI), tumour necrosis and age, may also affect prognosis 52.

The results of this study further affirm others' findings that identifying the mutational status is necessary. Mutational analysis has prognostic and predictive values and helps determine treatment type 49. The two patients with exon nine mutation (low response to standard-dose Imatinib) were identified, and treatment was adjusted accordingly.

Furthermore, this study presents the first mutational analysis of the KIT genes from Sudanese patients with GISTs. However, detecting PDGFRA and SDH genes for more samples would have empowered the study. Despite the few cases tested for exon 11 and exon 9 KIT mutations, they align with the international data on untreated GISTs showing that exon 11 and 9 mutations are mutually exclusive 59, 60.

One of the current study limitations is the small sample size. Additionally, since no Kaplan Meier estimators and/or log-rank tests are available, all conclusions and statistical considerations should be considered cautiously.

In conclusion, Sudanese patients with GISTs present late, and nearly half of them (≥47.2%) correspond to the malignant/high-risk category, hence needing TKI therapy. Most of the patients (72.2%) were from the different Sudan states other than Khartoum, and the highest prevalence was noted in those from western states (41.7%), suggesting some ethnic variability. Abdominal pain and swelling were the most frequent presenting symptoms. CT scan and upper/lower GI endoscopy were the most commonly used diagnostic procedures. The overall survival at 30 months means follow-up was 69.4%. Intraoperative documentation (presence of metastases, tumour rupture, same site) and the histopathology reporting are still suboptimal and need improvement to allow for reliable therapy decisions. TKI (Imatinib) needs a more critical approach to avoid unnecessary therapy.

## List of abbreviations

AFIP: Armed Forces Institute of Pathology
AJCC: American Joint Committee on Cancer
CT scan: Computed tomographic scan
DNA: Deoxyribonucleic acid
DOG1: Discovered on GIST1
ESMO: European Society for Medical Oncology
FFPE: Formalin-Fixed, Paraffin-Embedded
GIPAP: Glivec International Patient Assistance Program
GISTs: Gastrointestinal stromal tumours
H&E: Haematoxylin and Eosin
HPF: High power field
ICC: Intestinal Cell of Cajal
IHC: Immunohistochemistry
KIT: Tyrosine Kinase Inhibitor
KOH: Khartoum Oncology Hospital
MRI: Magnetic resonance imaging
NCCN: National Comprehensive Cancer Network
NIH: National Institute of Health
PDGFRA: Platelet-Derived Growth Factor Receptor Alpha
SDH: Succinate dehydrogenase
STI571: Selective tyrosine kinase inhibitor 571
TNM: Tumour, lymph node and metastasis
UK: United Kingdom
US: Ultrasound
USA: United States of America
